# Development of Highly Selective 1,2,3-Triazole-containing Peptidic Polo-like Kinase 1 Polo-box Domain-binding Inhibitors

**DOI:** 10.3390/molecules24081488

**Published:** 2019-04-16

**Authors:** Xue Zhi Zhao, Kohei Tsuji, David Hymel, Terrence R. Burke

**Affiliations:** Chemical Biology Laboratory, Center for Cancer Research, National Cancer Institute, National Institutes of Health, Frederick, MD 21702, USA; kohei.tsuji@nih.gov (K.T.); hymel.david@gmail.com (D.H.)

**Keywords:** Plk1, selectivity, polo-box domain, peptide, triazole

## Abstract

Members of the polo-like kinase (Plk) family of serine/threonine protein kinases play crucial roles in cell cycle regulation and proliferation. Of the five Plks (Plk1–5), Plk1 is recognized as an anticancer drug target. Plk1 contains multiple structural components that are important for its proper biological function. These include an N-terminal catalytic domain and a C-terminal non-catalytic polo-box domain (PBD). The PBD binds to phosphothreonine (pT) and phosphoserine-containing sequences. Blocking PBD-dependent interactions offers a potential means of down-regulating Plk1 function that is distinct from targeting its ATP-binding site. Previously, we demonstrated by tethering alkylphenyl chains from the *N*(π)-position of the His residue in the 5-mer PLHSpT, that we were able to access a hydrophobic “cryptic” binding pocket on the surface of the PBD, and in so doing enhance binding affinities by approximately 1000-fold. More recently, we optimized these PBD-ligand interactions using an oxime ligation-based strategy. Herein, using azide-alkyne cycloaddition reactions, we explore new triazole-containing PBD-binding antagonists. Some of these ligands retain the high PBD-binding affinity of the parent peptide, while showing desirable enhanced selectivity for the PBD of Plk1 relative to the PBDs of Plk2 and Plk3.

## 1. Introduction

Members of the polo-like kinase (Plk) family play crucial roles in mammalian cell cycle regulation and proliferation [1]. Proper function of Plks 1–4 requires the coordinated phosphorylation of serine and threonine residues by *N*-terminal kinase domains (KDs) as well as engagement of protein-protein interactions (PPIs) with phosphoserine (pS)/phosphothreonine (pT)-containing sequences by means of their *C*-terminal polo-box domains (PBDs) [2]. While Plks 1–3 share significant homology, Plk4 is more distantly related [3,4]. The association of Plk1 over-expression with neoplastic transformation and tumor aggressiveness has defined it as a potentially promising anticancer molecular target [5,6,7,8]. To date, issues of collateral cytotoxicity have arisen for Plk1 kinase inhibitors. This is in part due to a lack of selectivity arising from the general homology among kinase catalytic domains. Given the uniqueness of PBDs to the Plk family, targeting PBD-mediated PPIs may allow down-regulation of Plk1 function with greater kinome selectivity than with inhibitors directed at the KD. However, Plk2 and Plk3 have roles in checkpoint-mediated cell-cycle arrest and maintenance of genetic stability, and they may serve as potential tumor suppressors [3,4,9]. Therefore, in developing PBD-binding inhibitors, it is desirable that they are selective for Plk1 versus Plk2 and Plk3. Because of the high homology among the PBDs of Plks 1–3, achieving selectivity for the PBD of Plk1 presents an important and challenging objective [7,8,10,11,12,13].

**Figure 1:**
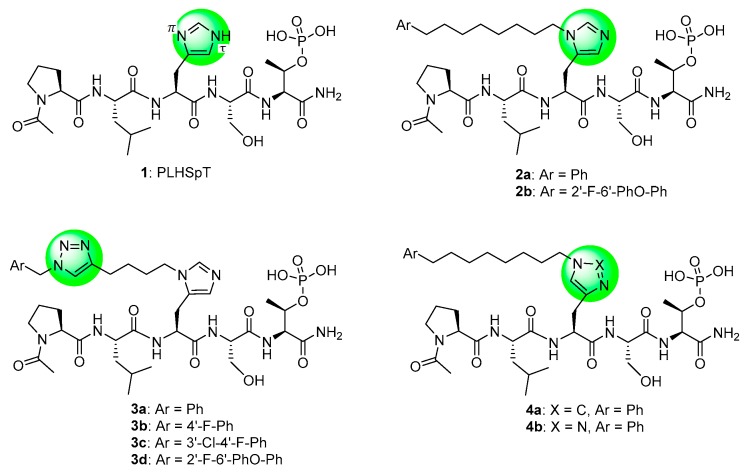
The polo-box interacting protein 1 (PBIP) pT78-derived peptide **1** and related derivatives discussed in the text.

In designing Plk1 PBD-binding inhibitors, we have previously started with the polo-box interacting protein 1 (PBIP1)-derived 5-mer PLHSpT (**1**) (Figure 1) [7,14]. We found that up to 1000-fold enhancement of Plk1 PBD-binding affinity can be achieved by appending alkylphenyl groups from the His *N*(π)-position (as exemplified by **2a**) [15]. A crystal structure of PBD-bound **2a** revealed that the alkylphenyl group is situated within a hydrophobic aromatic box defined by residues Y417, Y421, Y481, L478, F482 and Y485. This may be considered as being “cryptic” in nature, since it is revealed by rotation of the Y481 side chain [15]. We have reached the pocket from parent **1** by a variety of approaches, including tethering alkylphenyl groups from the Pro residue [16], an amino-terminal *N*-alkyl Gly residue [17] and from macrocyclic variants [18,19]. The pocket can also be accessed from more extended peptides, such as the amino-terminal Phe residue of the PBIP1-derived peptide FDPPLHSpTA [20,21,22].

The ability to engage the cryptic pocket has been a critical element of the highest affinity PBD-binding ligands reported to date. Within this context, the pT-2 position arguably represents the most efficient position from which to achieve this access, since it is the most proximal residue to the critical “SpT” recognition motif [23]. By examining a variety of non-proteinogenic amino acid residues at the pT-2 position, we found that the highest affinities were shown by those peptides having alkylation at the His-*N*(π) position, which provided approximately 50-fold higher affinity than alkylation at the isomeric His-*N*(τ)position (peptides **2a** and **4a**, respectively, Figure 1) [24]. Yet, optimizing these interactions has been made difficult due to the tediousness of preparing individual His-*N*(π)-alkyl analogs. In response to these challenges, we employed an oxime-based post-solid phase peptide diversification strategy that allowed us to screen more than 80 analogs. Ligands such as **2b** resulted, which show enhanced Plk1 PBD affinity or selectivity relative to parent **2a** [25,26]. 

The utility of 1,2,3-triazoles for introducing conformational constraint in peptidomimetic chemistry has been reported [27,28,29,30,31,32,33,34,35]. A triazole replacement of the imidazole ring in His has been used to prepare constrained His mimics [36]. More recently, triazole-based His mimetics bearing long-chain alkylphenyl groups have been examined within the context of non-peptidic Plk1 PBD inhibitors [37]. However, the best of these constructs showed Plk1 PBD-binding affinities that were 5- to 18-fold less potent than **1** (which itself exhibits 3-orders of magnitude less affinity than **2a**) [37]. Herein, we report the use of on-resin azide-alkyne cycloaddition reactions to introduce 1,2,3-triazole functionality into potent lead Plk1 PBD inhibitors based on **2a** [15] and **2b** [25,26]. The triazole rings were intended either to induce conformational constraint (**3a**–**3d**) or to serve as a His mimetic (**4b**). This work has allowed us to prepare in facile fashion, new ligands that retain the high Plk1 PBD-binding affinity of the parent peptide, while enhancing selectivity for the PBD of Plk1 relative to the PBDs of Plk2 and Plk3.

## 2. Results and Discussion

### 2.1. Synthesis

**Scheme 1:**
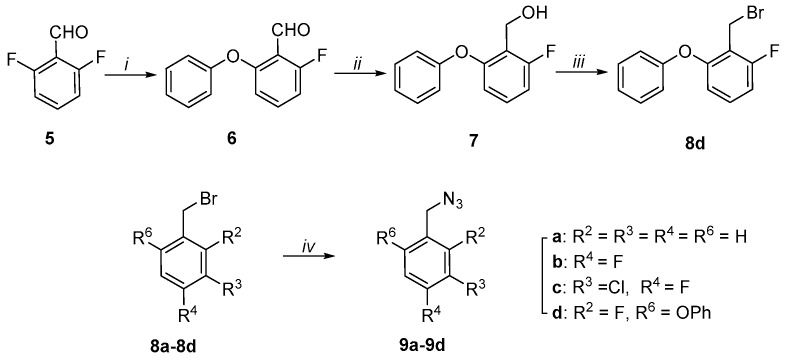
Preparation of azides **9a**–**9d**. *Reagents and Conditions: i*) PhOH, K_2_CO_3_, dimethylacetamide (DMA), 165 °C; *ii*) NaBH_4_, MeOH; *iii*) CBr_4_, PPh_3_, CH_3_CN; *iv*) NaN_3_, acetone:H_2_O (5:1).

Benzylazides **9a**–**9d** were prepared using S_N_2 reactions of sodium azide with commercially available benzyl bromides **8a**–**8c** and freshly prepared **8d** (Scheme 1). Bromide **8d** was synthesized in three steps from commercially available 2,6-difluorobenzylaldehyde (**5**). Displacement of one fluoro group in **5** by phenol afforded **6** [25], which was then reduced with sodium borohydride to yield alcohol **7**. Application of the Appel reaction [38] using carbon tetrabromide and triphenylphosphine afforded bromide **8d** (Scheme 1). 

**Scheme 2:**
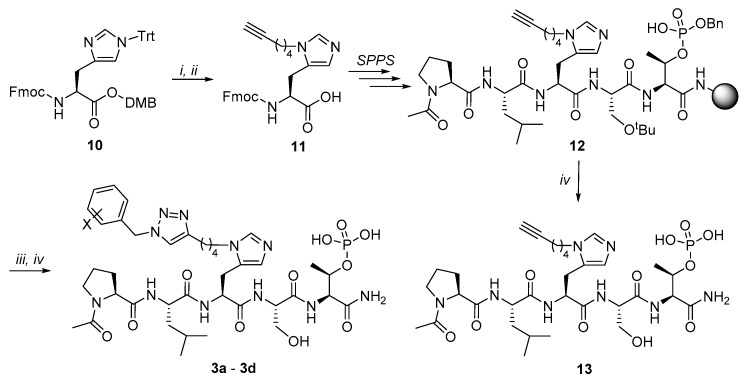
Preparation of triazole-containing peptides **3a**–**3d** and alkyne-labeled peptide **13**. *Reagents and Conditions: i*) CH≡C(CH_2_)_4_OH, trifluoromethanesulfonic anhydride (Tf_2_O), *N*,*N*-diisopropylethylamine (DIEA), dichloromethane (DCM); *ii*) trifluoroacetic acid (TFA), triisopropylsilane (TIS); *iii*) Bn-N_3_ (**9a**–**9d**), CuI, l-ascorbic acid, dimethylformamide (DMF):BuOH:Pyr (3:5:2); *iv*) TFA:H_2_O:TIS (95:2.5:2.5).

The protected *N*(π)-alkyne-labeled His derivative **11** was easily obtained by alkylating *N^α^*-Fmoc-*N^τ^*-Trt-l-His 2,4-dimethoxylbenzyl ester (**10**) [25] with hex-5-yn-1-ol according to our previously reported methodology [39]. This was then used to prepare the fully protected alkyne-containing peptide **12** on NovaSyn^®^ TGR resin by standard Fmoc solid-phase peptide synthesis (SPPS) protocols in *N*-methylpyrrolidone (NMP) (Scheme 2). Resin **12** was subsequently subjected to Cu(I) catalyzed [3 + 2] cycloaddition reactions (on-resin CuAAC) with the related benzylazides **9a**–**9d** (Scheme 2). The regioselective CuAAC reaction has been an important advance that provides a reliable means for selectively assembling 1,4-disubstituted 1,2,3-triazoles [40,41,42]. The resins were cleaved using a cocktail solution of TFA:H_2_O:triisopropylsilane (TIS) (95:2.5:2.5) to provide the triazole-containing peptides **3a**–**3d** following HPLC purification. Alkyne-labeled peptide **13** was prepared by cleavage of **12** directly.

**Scheme 3:**
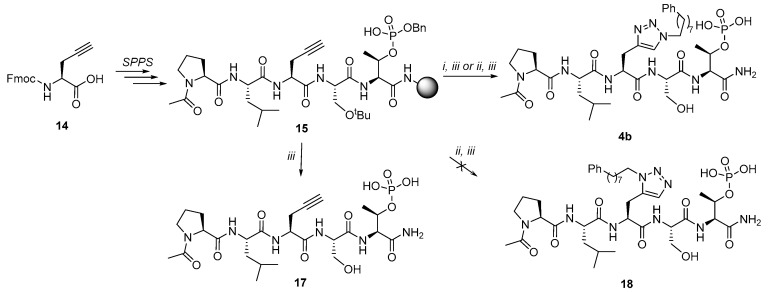
Preparation of triazole-containing peptides **4b** and alkyne-labeled peptide **17**. *Reagents and Conditions: i*) Ph(CH_2_)_8_N_3_ (**16**), CuI, l-ascorbic acid, DMF:BuOH:Pyr (3:5:2); *ii*) Cp*RuCl(PPh_3_)_2_, Ph(CH_2_)_8_N_3_ (**16**), DMF; *iii*) TFA:H_2_O:TIS (95:2.5:2.5).

Preparation of the triazole-based His mimetic containing peptide **4b** and alkyne-labeled peptide **17** are shown in Scheme 3. Protected **15** was prepared on NovaSyn^®^ TGR resin using commercially available Fmoc-l-propargylglycine (**14**) and standard Fmoc SPPS protocols. Cleavage of resin **15** and HPLC purification yielded alkyne-containing peptide **17** directly. Alternatively, an on-resin CuAAC reaction of **15** with phenyloctylazide **16** followed by resin cleavage gave peptide **4b** after HPLC purification (Scheme 3).

It was our original intent to prepare both 1,4-substituted and 1,5-substituted triazoles as mimetics of the isomeric *N*(τ)- and *N*(π)-alkylated His analogs, respectively. As stated above, the CuAAC reaction provides a reliable means for selectively assembling 1,4-disubstituted 1,2,3-triazoles [40,41,42]. Accordingly, when we subjected resin-bound alkyne-containing peptide **15** to CuAAC-catalyzed cycloaddition with phenyloctylazide **16**, we obtained a peptide following resin cleavage and HPLC purification, whose structure we assigned as **4b** (Scheme 3). Alternatively, the ruthenium-catalyzed cycloaddition of azides with alkynes (RuAAC) has been reported to regioselectively yield 1,5-disubstituted 1,2,3-triazoles [43,44]. Based on this, we used the on-resin RuAAC-catalyzed [3 + 2] cycloaddition reaction of the alkyne group of resin **15** and azide **16** with the expectation of obtaining the isomeric 1,5-substituted triazole **18** (Scheme 3). However, the resulting peptide was identical in all respects with peptide **4b** (^1^H-, ^13^C- and ^31^P-NMR). At this point, a search of the literature revealed that a simple method has been reported, which permits reliable establishment of triazole regio-substitution based on chemical shifts in one-dimensional ^13^C-NMR spectra [45]. The C5 signal of 1,4-disubstituted-1*H*-1,2,3-triazoles characteristically appears at approximately δ = 120 ppm, while the C4 signal of 1,5-disubstituted-1*H*-1,2,3-triazoles is usually found at δ = 133 ppm. In our case, the products obtained from both CuAAC and RuAAC chemistries provided a diagnostic signal of δ = 123.04 ppm, indicating that the 1,4-substituted triazole (**4b**) was obtained in both cases.

### 2.2. Biological Evaluation

We employed fluorescence polarization (FP) assays to evaluate binding affinities against the isolated PBDs of Plk1, Plk2 and Plk3 (Table 1, Figure S1 in Supplementary Material). Compared with the parent peptide **1** (IC_50_ = 650 nM) replacement of the His imidazole ring with an alkyne group resulted in an approximate 2-fold loss of Plk1 PBD-binding affinity (**17**, IC_50_ = 1000 nM, Table 1). Interestingly, peptide **13** (IC_50_ = 1100 nM) showed equivalent Plk1 PBD-binding affinity, in spite of the fact that it included a hex-5-yn-1-yl moiety at the His *N*(π)-position. This group would be expected to partially engage the hydrophobic channel leading to the cryptic pocket. We have previously shown that peptides **2a** and **2b** exhibit binding affinities (IC_50_ values of approximately 15 nM) that are significantly more potent than parent peptide **1** (approximately 650 nM) [25,26]. Peptides **2a** and **2b** show more than 10-fold selectivity for Plk1 relative to Plk2 and approximately 70-fold and 20-fold over Plk3, respectively (Table 1).

Peptides **3a**–**3d** represent a series of analogs having 1,4-substituted triazoles tethered from the His *N*(π)-position by –(CH_2_)_4_– chains. Similar to **2a** and **2b**, this results in a total chain extension of 8-units between the His *N*(π)-nitrogen and the terminal aryl group. We had previously shown that this is an optimal length by examining a series of sequentially lengthened tethers [15]. Introducing a 4-fluoro substituent (**3b**, IC_50_ = 100 nM, Table 1) or 3-chloro-4-fluoro substituents (**3c**, IC_50_ = 130 nM, Table 1) were intended to potentially enhance interactions with the hydrophobic cryptic pocket. However, these did not significantly alter affinity relative to **3a**. The reasons for this are not clear. Although peptide **3a** was designed to mimic peptide **2a**, it shows an approximate 8-fold relative loss of Plk1 PBD-binding affinity (**3a**, IC_50_ = 110 nM, Table 1). In contrast to the marked loss of affinity incurred by introducing the triazole ring to **2a**, the triazole-containing mimetic of **2b** showed good retention of Plk1 PBD-binding (**3d**, IC_50_ = 25 nM). While peptides **3a**–**3c** contain a single tethered phenyl ring, peptide **3d** has a bis-aryl system. The greater extension afforded by this latter arrangement may permit better retention of binding interactions with the cryptic pocket than is afforded by peptides have a single phenyl ring. Importantly, **3d** showed extremely high selectivity for Plk1 relative to Plk2 (IC_50_ = 5900 nM) and Plk3 (IC_50_ = 9900 nM) (Table 1).

In contrast to peptides **3a**–**3d**, where triazole rings were inserted into His-*N*(π)-tethered chains to potentially induce conformational constraint proximal to the cryptic binding pocket, peptide **4b** represents a triazole mimetic of the His imidazole ring. In our previous efforts to access the cryptic pocket using a variety of amino acid derivatives at the pT-2 position, the highest affinities were obtained using His residues, with alkylation at the His-*N*(π) position (**2a**, Figure 1) being significantly preferred to alkylation at the isomeric His-*N*(τ) position (**4a**, Figure 1) [24]. In spite of the fact that the 1,4-triazole substitution pattern of **4b** does not appear to optimally replicate the geometry of the His-*N*(π)-1,2-imidazole pattern shown by **2a**, its Plk1 binding affinity (IC_50_ = 17 nM) equals that of **2a** (Table 1). The Plk1 PBD selectivity of **4b** is slightly better than **2a** against the Plk2 and Plk3 PBDs (690 nM and 3400 nM, respectively).

It is known that auto-inhibitory interdomain interactions between the KD and PBD can result in decreased potencies in assays that employ full-length Plk1 relative to assays that use isolated PBD preparations, which lack a KD component [23]. The selectivity data shown in Table 1 were obtained using fluorescence polarization assays with isolated PBDs. In contrast, Table 2 shows binding data from an ELISA assay employing full-length Plk1 (Figure S2 in Supplementary Material). Peptides possessing His-*N*(π)-tethered chains showed an approximate order-of-magnitude potency reduction in the full-length assay relative to the isolated PBD assay (27-fold and 19-fold reductions for **2a** and **2b**, respectively). However, triazole-containing peptides experienced significantly greater losses of inhibitory potency (160-fold for **4b** and 480-fold for **3d**). The larger differences may indicate a reduced ability of these peptides to effectively relieve auto-inhibition or to engage the PBD cryptic pocket in the full-length construct. 

Although there are no crystals structure of full-length Plk1, which might clarify the mechanisms of autoinhibition, a co-crystal structure of Map205-stabilized isolated Plk1 KD and PBD has been solved (PDB accession code: 4J7B) [46]. In this structure the KD is situated on the face of the PBD opposite the phosphopeptide-binding site. In such an orientation, the KD displaces downward an extended loop of the PBD (residues 490–510) from where it is typically observed in isolated PBD crystal structures with bound phosphopeptides. This conformational change prevents the loop from participating in an extensive network of water-mediated hydrogen bonds with the peptide phosphate group. This may be related to the ability of the KD to inhibit ligand binding to the PBD in full-length Plk1. It is unclear from this how access to the cryptic pocket would be adversely impacted in full-length Plk1 or why the triazole-containing peptides would be more sensitive to these effects. However, it is intriguing that this loop originates from the αB helix (residues 470–489), which forms an important component of the cryptic binding pocket.

## 3. Experimental Section

### 3.1. Synthesis

#### 3.1.1. General Procedures

As previously reported [26], proton (^1^H) and carbon (^13^C) NMR spectra were recorded on a Varian 400 MHz spectrometer or a Varian 500 MHz spectrometer (Varian, Palo Alto, CA, USA) and are reported in ppm relative to tetramethylsilane (TMS) and referenced to the solvent in which the spectra were collected. Solvent was removed by rotary evaporation under reduced pressure and anhydrous solvents were obtained commercially and used without further drying. Purification by silica gel chromatography was performed using Combiflash instruments (Telenyde ISCO, Lincoln, NE, USA) with EtOAc-hexanes or CH_2_Cl_2_-MeOH solvent systems. Preparative high pressure liquid chromatography (HPLC) was conducted using a Waters Prep LC4000 system (Waters, Milford, MA, USA) having photodiode array detection and C18 columns (catalogue No. 00G4436-P0-AX, 250 mm × 21.2 mm 10 μm particle size, 110 Å pore, Phenomenex, Torrance, CA, USA) at a flow rate of 10 mL/min. Binary solvent systems consisting of A = 0.1% aqueous TFA and B = 0.1% TFA in acetonitrile were employed with gradients as indicated. Products were obtained as amorphous solids following lyophilization. Electrospray ionization-mass spectra (ESI-MS) were acquired with an Agilent LC/MSD system (Agilent, Santa Clara, CA, USA) equipped with a multimode ion source. High resolution mass spectrometric (HRMS, ThermoFisher Scientific, Grand Island, NY, USA) were acquired by LC/MS-ESI with a LTQ-Orbitrap-XL at 30 K resolution.

#### 3.1.2. Synthesis of 2-Fluoro-6-phenoxybenzaldehyde (**6**)

According to the literatures [25,47], to a solution of 2,6-difluorobenzaldehyde (**5**) (11 mL, 102 mmol) and phenol (9.6 g, 102 mmol) in dimethylacetamide (DMA) (50 mL) was added potassium carbonate (14 g, 102 mmol) and the mixture was heated and refluxed (165 °C, 2 h). The mixture was cooled to room temperature, diluted with H_2_O (100 mL), extracted with CH_2_Cl_2_ and the combined organic extract was dried (Na_2_SO_4_) and concentrated. The resulting residue was purified by silica gel chromatography to afford product **6** as a colorless oil (14.1 g, 64% yield). ^1^H-NMR (400 MHz, CDCl_3_) δ 10.52 (s, 1H), 7.47–7.40 (m, 3H), 7.23 (t, *J* = 7.4 Hz, 1H), 7.09 (dd, *J* = 8.6, 1.2 Hz, 2H), 6.89–6.85 (m, 1H), 6.66 (d, *J* = 8.5 Hz, 1H). ^13^C-NMR (101 MHz, CDCl_3_) δ 186.79 (1C, d, *J* = 2.3 Hz), 162.90 (1C, d, *J* = 263.4 Hz), 160.50 (1C, d, *J* = 5.2 Hz), 155.63, 135.73 (1C, d, *J* = 11.6 Hz), 130.19 (2C), 124.90, 119.85 (2C), 116.03 (1C, d, *J* = 9.5 Hz), 113.48 (1C, d, *J* = 3.7 Hz), 110.81 (d, *J* = 21.2 Hz). ESI-MS *m*/*z*: 239.0 [M + Na^+^]. 

#### 3.1.3. Synthesis of (2-Fluoro-6-phenoxyphenyl)methanol (**7**) 

To a solution of 2-fluoro-6-phenoxybenzaldehyde (**6**) (5.9 g, 27 mmol) in MeOH (100 mL) was added sodium borohydride (1.0 g, 27 mmol) portion-wise at 0 °C and the mixture was stirred (0 °C, 30 min), then concentrated. The resulting residue was partitioned between EtOAc and brine, dried (Na_2_SO_4_), concentrated and purified by silica gel chromatography to afford product **7** as a colorless oil (5.7 g, 96% yield). ^1^H-NMR (500 MHz, CDCl_3_) δ 7.40–7.37 (m, 2H), 7.23–7.16 (m, 2H), 7.06–7.04 (m, 2H), 6.86 (t, *J* = 8.9 Hz, 1H), 6.66 (d, *J* = 8.3 Hz, 1H), 4.85 (s, 2H).^13^C-NMR (126 MHz, CDCl_3_) δ 161.70 (1C, d, *J* = 247.3 Hz), 156.95 (1C, d, *J* = 7.4 Hz), 156.62, 129.98 (2C), 129.57 (1C, d, *J* = 10.5 Hz), 124.02, 119.41 (1C, d, *J* = 18.0 Hz), 119.04 (2C), 113.77 (1C, d, *J* = 3.3 Hz), 110.48 (1C, d, *J* = 22.6 Hz), 54.02 (1C, d, *J* = 5.1 Hz).

#### 3.1.4. Synthesis of 2-(Bromomethyl)-1-fluoro-3-phenoxybenzene (**8d**)

According to the literature [38], triphenylphosphine (10 g, 39 mmol) was added to a solution of (2-fluoro-6-phenoxyphenyl)-methanol (**7**) (5.7 g, 26 mmol) in acetonitrile (70 mL) and the suspension was cooled to 0 °C and carbon tetrabromide (13 g, 39 mmol) was added. The suspension turned to a clear brown solution and then to a white suspension after 2 min. The reaction suspension was stirred (room temperature, 30 min), then diluted with EtOAc and the organic phase was concentrated and purified by silica gel chromatography to provide product **8d** as a colorless oil (7.4 g, 99% yield). ^1^H-NMR (500 MHz, CDCl_3_) δ 7.43–7.40 (m, 2H), 7.23–7.19 (m, 2H), 7.12–7.09 (m, 2H), 6.86 (t, *J* = 9.2 Hz, 1H), 6.63 (d, *J* = 8.4 Hz, 1H), 4.70 (d, *J* = 1.3 Hz, 2H). ^13^C-NMR (126 MHz, CDCl_3_) δ 161.56 (1C, d, *J* = 250.5 Hz), 156.91 (1C, d, *J* = 6.4 Hz), 156.34, 130.16 (1C, d, *J* = 10.5 Hz), 129.98 (2C), 124.30, 119.61 (2C), 117.10 (1C, d, *J* = 17.2 Hz), 113.42 (1C, d, *J* = 3.2 Hz), 110.11 (1C, d, *J* = 21.6 Hz), 20.10 (1C, d, *J* = 5.4 Hz).

#### 3.1.5. General Procedure A for the Synthesis of Azides **9a**–**9d** and **16**

To a solution of bromides **8a**–**8d** or commercially available (8-bromooctyl)benzene (7.0 mmol) in acetone (10 mL) and H_2_O (2.0 mL) was added sodium azide (1.8 g, 28 mmol) and the mixture was stirred (55 °C, 15 h). The reaction was quenched by the addition of H_2_O, extracted with Et_2_O and the combined organic phase was washed with brine, dried (Na_2_SO_4_), concentrated and purified by silica gel chromatography to provide the target azides **9a**–**9d,** and **16**.

#### 3.1.6. Synthesis of (Azidomethyl)benzene (**9a**) 

According to the literature [48], treatment of (bromomethyl)benzene (**8a**) [48] as outlined in general procedure A provided title compound **9a** as a colorless oil (57% yield). ESI-MS *m*/*z*: 106.1 (MH^+^ − N_2_).

#### 3.1.7. Synthesis of 1-(Azidomethyl)-4-fluorobenzene (**9b**)

Treatment of 1-(bromomethyl)-4-fluorobenzene (**8b**) as outlined in general procedure A provided the title compound **9b** as a colorless oil (82% yield). ^1^H-NMR (500 MHz, CDCl_3_) δ 7.32 (dd, *J* = 8.4, 5.4 Hz, 2H), 7.10 (t, *J* = 8.6 Hz, 2H), 4.34 (s, 2H). ESI-MS *m*/*z*: 124.1 (MH^+^ − N_2_).

#### 3.1.8. Synthesis of 4-(Azidomethyl)-2-chloro-1-fluorobenzene (**9c**)

Treatment of 4-(bromomethyl)-2-chloro-1-fluorobenzene (**8c**) as outlined in general procedure A provided the title compound **9c** as a colorless oil (49% yield). ^1^H-NMR (500 MHz, CDCl_3_) δ 7.40 (dd, *J* = 6.9, 2.1 Hz, 1H), 7.22 (ddd, *J* = 7.0, 4.7, 2.1 Hz, 1H), 7.18 (t, *J* = 8.5 Hz, 1H), 4.34 (s, 2H). ^13^C-NMR (126 MHz, CDCl_3_) δ157.94 (d, *J* = 249.9 Hz), 132.53 (d, *J* = 4.0 Hz), 130.37, 127.85 (d, *J* = 7.3 Hz), 121.46 (d, *J* = 18.0 Hz), 116.96 (d, *J* = 21.4 Hz), 53.54. ESI-MS *m*/*z*: 158.1 (MH^+^ − N_2_)

#### 3.1.9. Synthesis of 2-(Azidomethyl)-1-fluoro-3-phenoxybenzene (**9d**)

Treatment of 2-(bromomethyl)-1-fluoro-3-phenoxybenzene (**8d**) as outlined in general procedure A provided the title compound **9d** as a colorless oil (58% yield). ^1^H-NMR (400 MHz, CDCl_3_) δ 7.79 (t, *J* = 7.9 Hz, 2H), 7.67–7.63 (m, 1H), 7.61–7.57 (m, 1H), 7.47–7.45 (m, 2H), 7.28 (t, *J* = 8.6 Hz, 1H), 7.05 (d, *J* = 8.4 Hz, 1H), 4.91 (s, 2H). ^13^C-NMR (101 MHz, CDCl_3_) δ 162.04 (d, *J* = 248.7 Hz), 157.26 (d, *J* = 7.0 Hz), 156.14, 130.29 (d, *J* = 10.5 Hz), 130.00 (2C), 124.30, 119.47 (2C), 114.17 (d, *J* = 18.2 Hz), 113.12 (d, *J* = 3.3 Hz), 110.09 (d, *J* = 22.2 Hz), 42.55 (d, *J* = 4.0 Hz). ESI-MS *m*/*z*: 216.1 (MH^+^ − N_2_).

#### 3.1.10. Synthesis of (8-Azidooctyl)benzene (**16**)

Treatment of commercially available (8-bromooctyl)benzene as outlined in general procedure A provided the title compound **16** as a colorless oil (68% yield). ^1^H-NMR (400 MHz, CDCl_3_) δ 7.32–7.28 (m, 2H), 7.22–7.18 (m, 3H), 3.28 (t, *J* = 7.0 Hz, 2H), 2.64 (t, *J* = 7.7 Hz, 2H), 1.66–1.58 (m, 4H), 1.41–1.33 (m, 8H). ^13^C-NMR (101 MHz, CDCl_3_) δ 142.82, 128.39 (2C), 128.23 (2C), 125.59, 51.49, 35.96, 31.45, 29.34, 29.19, 29.08, 28.84, 26.71. ESI-MS *m*/*z*: 204.2 (MH^+^ − N_2_).

#### 3.1.11. Synthesis of *N*^α^-(((9*H*-Fluoren-9-yl)methoxy)carbonyl)-*N*^π^-(hex-5-yn-1-yl)-l-histidine (**11**) 

As previously reported [39], to a solution of trifluoromethanesulfonic anhydride (2.8 mL, 2.8 mmol) in CH_2_Cl_2_ (5.0 mL) was added a solution of hex-5-yn-1-ol (0.31 mL, 2.8 mmol) and *N*-ethyl-*N*-isopropylpropan-2-amine (0.49 mL, 2.8 mmol, 1.0 M in CH_2_Cl_2_) in CH_2_Cl_2_ (10 mL) dropwise under argon at −78 °C and the mixture was stirred at −78 °C (20 min). To this was added a solution of (*S*)-2,4-dimethoxybenzyl 2-((((9*H*-fluoren-9-yl)methoxy)carbonyl)amino)-3-(1-trityl-1*H*-imidazol-4-yl)propanoate (**10**) [24] (2.0 g, 2.5 mmol) in CH_2_Cl_2_ (5.0 mL) at −78 °C and the mixture was allowed to come to room temperature and stirred (overnight). The solvent was removed by evaporation and a solution of TFA:TIS (10:1, 10 mL) was added and the mixture was stirred at room temperature (2 h). The reaction mixture was concentrated and the resulting residue was purified by silica gel chromatography to provide the title compound **11** as a colorless sticky oil (0.53 g, 46% yield). ESI-MS *m/z*: 458.2 (MH^+^).

### 3.2. Peptide Synthesis

#### 3.2.1. General Solid-Phase Peptide Synthesis (SPPS)

As previously reported [26], the protected amino acids used were Fmoc-l-Thr(PO(OBzl)OH)-OH, Fmoc-l-Ser(O*^t^*Bu)-OH, Fmoc-l-Leu-OH, and Fmoc-l-Pro-OH (purchased from Novabiochem, MilliporeSigma, Burlington, MA, USA). Peptides were synthesized on a NovaSyn^®^ TGR resin (Novabiochem Cat#. 855009) using standard Fmoc SPPS protocols in *N*-methyl-2-pyrrolidone (NMP). Coupling reagents used were 1-[bis(dimethylamino)methylene]-1*H*-1,2,3-triazolo[4,5-b]pyridinium 3-oxid hexafluorophosphate (HATU) (5.0 equivalents) and *N*,*N*-diisopropylethylamine (DIPEA) (10 equivalents). Non-coded amino acid residues were coupled using 2.5 equivalents amino acids. Deprotection was performed using 20% piperidine in DMF (15 min, twice). Amino-terminal acetylation was performed using 1-acetylimidazole. Finished resins were washed with NMP, MeOH, CH_2_Cl_2_, and Et_2_O, dried under vacuum and cleaved by treatment with a solution of TFA:H_2_O:TIS (95:2.5:2.5) (5 h). The resin was removed by filtration, and the filtrate was concentrated under vacuum and the resulting residue was dissolved in 50% aqueous acetonitrile (5 mL) and purified by reverse-phase HPLC as outline above in General Synthetic Procedures.

#### 3.2.2. Synthesis of Peptides **3a**–**3d** and **4b** Using On-resin Copper-catalyzed Alkyne-azide Cycloaddition Reaction (CuAAC)

By sequential coupling, Fmoc-Thr(PO(OBzl)OH)-OH, Fmoc-Ser(O*^t^*Bu)-OH, alkynyl-labelled *N(*π*)*-alkylated Fmoc-His-OH (**11**) or Fmoc-l-propargylglycine (**14**), Fmoc-Leu-OH, and Fmoc-Pro-OH were loaded onto NovaSyn^®^ TGR resin using the general SPPS protocols outlined above. The pre-loaded resin (**12** or **15**, 0.02 mmol) was mixed with a solution of azides (**9a**–**9d** or **16**) (0.14 mmol), copper(I) iodide (0.26 mmol), DIEA (0.34 mmol) and l-ascorbic acid (0.14 mmol) in BuOH:DMF:pyridine (5:3:2, 1.5 mL) and the mixture was stirred (17 h). The resin was washed with NMP, MeOH, CH_2_Cl_2_, and Et_2_O, dried under vacuum and then cleaved by treatment with a solution of TFA:H_2_O:TIS (95:2.5:2.5) (5 h). The resin was removed by filtration and the filtrate was concentrated under vacuum and the resulting residue was subjected to preparative HPLC purification.

#### 3.2.3. Synthesis of Peptide **4b** Using On-resin Ruthenium-catalyzed Alkyne-azide Cycloaddition Reaction (RuAAC) 

According to literature [36,49], pre-loaded alkyne-labeled NovaSyn^®^ TGR resin **15** (0.049 mmol) was mixed with Cp*RuCl(PPh_3_)_2_ [pentamethylcyclopentadienylbis(triphenylphosphine) ruthenium (II) chloride] (0.049 mmol) in DMF (1.0 mL) at room temperature (1 h). A solution of (8-azidooctyl)benzene (**16**) (56 mg, 0.24 mmol) in DMF (1.0 mL) was added and the mixture was stirred at room temperature (24 h). The resulting dark brown resin was washed with NMP, MeOH, CH_2_Cl_2_, and Et_2_O, dried under vacuum and cleaved by treatment with a solution of TFA:H_2_O:TIS (95:2.5:2.5) (5 h). The resin was removed by filtration and the filtrate was concentrated under vacuum and the resulting residue was purified by preparative HPLC.

#### 3.2.4. Peptide Data

*Peptide***3a**. Linear gradient of 0% B to 80% B over 30 min, retention time = 16.0 min. ESI-MS *m*/*z*: 888.4 (MH^+^). HRMS calcd C_39_H_59_N_11_O_11_P(MH^+^), 888.4128; found, 888.4139.

*Peptide***3b**. Linear gradient of 0% B to 80% B over 30 min, retention time = 16.4 min. ESI-MS *m*/*z*: 906.4 (MH^+^). HRMS calcd C_39_H_58_FN_11_O_11_P (MH^+^), 906.4033; found, 906.4045.

*Peptide***3c**. Linear gradient of 0% B to 80% B over 30 min, retention time = 17.0 min. ESI-MS *m*/*z*: 940.3 (MH^+^). HRMS calcd C_39_H_57_ClFN_11_O_11_P (MH^+^), 940.3644; found, 940.3650.

*Peptide***3d**. Linear gradient of 0% B to 80% B over 30 min, retention time = 17.8 min. ESI-MS *m*/*z*: 998.2 (MH^+^). HRMS calcd C_45_H_62_FN_11_O_12_P(MH^+^), 998.4296; found, 998.4311.

*Peptide***4b**. Linear gradient of 0% B to 80% B over 30 min, retention time = 22.6 min. ESI-MS *m*/*z*: 864.4 (MH^+^). HRMS calcd C_39_H_63_N_9_O_11_P (MH^+^): 864.4379; found, 864.4365.

*Peptide***13**. Linear gradient of 0% B to 80% B over 30 min, retention time = 14.8 min. ESI-MS *m*/*z*: 755.3 (MH^+^). HRMS calcd C_32_H_52_N_8_O_11_P (MH^+^): 755.3488; found, 755.3497.

*Peptide***17**. Linear gradient of 0% B to 80% B over 30 min, retention time = 15.0 min. ESI-MS *m*/*z*: 633.2 (MH^+^). HRMS calcd C_25_H_42_N_6_O_11_P (MH^+^), 633.2644; found, 633.2625.

### 3.3. Determination of Binding Selectivity against the PBDs of Plks 1–3 Using Fluorescence Polarization

#### 3.3.1. Expression and Purification of Isolated PBDs of Plks 1–3 for Fluorescence Polarization Assays

As previously reported [25,26,50], a plasmid encoding myc-tagged Plk1 PBD was purchased from Addgene (Plasmid #41162, Watertown, MA, USA) [51]. Plasmids encoding the myc-tagged PBDs of Plk2 and Plk3 were generous gifts from Prof. Erich Nigg (Univ. of Basel, Basel, Switzerland) [51]. ~20 M HEK (human embryonic kidney)-293T cells (2 × 15 cm plates) were transfected with each plasmid using TurboFect reagent. Following 24 h expression, cells were harvested, lysed in buffer [phosphate buffered saline (PBS, pH 7.4) containing 0.5% NP-40 and protease/phosphatase inhibitor cocktail] using freeze/thaw cycles (3×) and centrifuged at 12,500× *g* for 10 min at 4 °C. The supernatant containing expressed protein was diluted into 8 mL of PBS (pH 7.4) containing protease/phosphatase inhibitor cocktail. This protein solution was added to a 1 mL bed of myc-agarose resin (Thermo Scientific, Waltham, MA, USA) and allowed to bind for 2 h at 4 °C with gentle rotation. The lysate was eluted and the resin was washed 4× with 4-(2-hydroxyethyl)-1-piperazineethanesulfonic acid (HEPES) buffered saline (HBS) containing 0.05% Tween-20, 1 mM dithiothreitol (DTT) and 1 mM ethylenediaminetetraacetic acid (EDTA). The bound PBD protein was then eluted with a 1 mg/mL solution of myc peptide (EQKLISEEDL) in HBS + 1 mM DTT and 1 mM EDTA. The purified PBD protein was dialyzed 5× with HBS + 1 mM DTT and 1 mM EDTA using a 10 kDa molecular weight cut-off (MWCO) filter (Sigma-Aldrich, St. Louis, MO, USA) fixed angle rotor at 7500× *g*, 4 °C, 10 min). The concentration of the final protein solution was determined by absorbance at 280 nm and purity was determined by SDS-PAGE (sodium dodecyl sulfate-polyacrylamide gel electrophoresis) with Coomassie staining.

#### 3.3.2. Evaluation of Binding Affinities against the Isolated PBDs of Plk1, Plk2 and Plk3 Using Fluorescence Polarization Assays

As previously reported [25,26,50], isolated PBD protein was diluted to a 2× working dilution in assay buffer (HEPES-buffered saline with 0.05% Tween-20, 1 mM DTT, and 1 mM EDTA). The following final protein concentrations used were: 80 nM for Plk1 PBD; 80 nM for Plk2 PBD and 130 nM for Plk3 PBD. These concentrations represent the approximate K_d_ values determined for the respective fluorescence polarization probe sequences. Inhibitors were serially diluted to generate 4× working dilutions in assay buffer containing 4% DMSO. 20 μL of 2× PBD solution was added to each well of a 384-well plate (0% binding controls received 20 μL of assay buffer). 10 μL of the 4× inhibitor solution (or DMSO blank) was added to corresponding wells and allowed to pre-incubate at RT for 30 min with shaking. The following sequences were utilized as fluorescent probes: 5CF-GPMQSpTPLNG-NH_2_ for Plk1 PBD; 5CF-GPMQTSpTPKNG-NH_2_ for Plk2 PBD and 5CF-PLATSpTPKNG-NH_2_ for Plk3 PBD [10]. Fluorescent probes were diluted to 40 nM (4×) in assay buffer and then 10 μL was added to each well. The plate was allowed to equilibrate at room temperature for (30 min) with shaking. The FP was read using a BioTek Synergy 2 plate reader (BioTek, Winooski, VT, USA) with 485/20 excitation and 528/20 emission. The FP values were obtained in triplicate and normalized to 100% (no inhibitor) and 0% binding (no protein) controls. Normalized values were plotted versus concentration and analyzed using non-linear regression in GraphPad Prism 8 (GraphPad Software, San Diego, CA, USA) [log(inhibitor) vs. response-variable slope (four parameter) model]. IC_50_ values are presented in Table 1 and represent average ± standard error of the mean (SEM).

### 3.4. Evaluation of Binding Affinities against Full-length Plk1 Using ELISA Assays

#### 3.4.1. Lysate Production for ELISA-based Inhibition Assay against Full Length Plk1

Assays were conducted as previously reported [25,26,50]. To summarize, a plasmid encoding myc-tagged full-length Plk1 (Addgene, Plasmid #41160) [52] was transiently transfected into HEK (human embryonic kidney)-293T cells using the TurboFect reagent (Thermo Scientific, Waltham, MA, USA) according to manufacturer’s instructions. Following 48 h expression, cells were harvested, lysed in buffer (PBS, pH 7.4 with 0.5% NP-40 and protease/phosphatase inhibitor cocktail) using freeze/thaw cycles (3×) and centrifuged at 10,000× *g* for 10 min at 4 °C. The supernatant was removed to provide a crude cytosolic lysate containing overexpressed, myc-tagged Plk1 (total protein concentration determined by bicinchoninic acid (BCA) assay).

#### 3.4.2. Determination of Inhibitory Potency in an ELISA Assay Using Full-Length Plk1

As previously reported [25,26,50] a biotinylated phosphopeptide (sequence: Biotin-Ahx-PMQS(pT)PLN-NH_2_) was diluted to 1 µM (from a 2 mM DMSO stock solution) in PBS (pH 7.4) and loaded onto the wells of a 96-well Neutravidin-coated plate (Pierce Biotechnology, ThermoFisher Scientific, Waltham, MA, USA) at 100 µL per well for 1 h (background control contained no biotinylated peptide). The wells were washed once with 150 µL PBST (PBS, pH 7.4 + 0.05% Tween-20), and then 100 µL of 1% BSA in PBS (pH 7.4) (blocking buffer) was added for 1 h. A cytosolic lysate-containing transiently expressed myc-tagged Plk1 protein was diluted to 300 µg/mL in PBS (pH 7.4) containing protease/phosphatase inhibitors (Pierce Biotechnology), mixed with competitive inhibitor (from a 10× stock in ~4% DMSO/PBS), and allowed to pre-incubate for 1 h (100 µL per well in a 96-well plate, 30 µg total protein). The blocked ELISA plate was washed 2× with PBST (PBS, pH 7.4 + 0.05% Tween-20) (150 µL) and the pre-incubated lysates were added to the plate and incubated (1 h). The wells were washed 4× with PBST (150 µL) and then treated with anti-myc primary antibody (1:1500 dilution in PBS, mouse monoclonal, Pierce Biotechnology) for 1 h. The wells were then washed 4× with PBST (150 µL), and incubated with rabbit anti-mouse horseradish peroxidase (HRP) conjugate [1:3000 dilution in 1% (%*w*/*v*) BSA in PBS, Pierce Biotechnology] for 1 h. The wells were then washed 5× with PBST (150 µL) and incubated with Turbo TMB (3,3′,5,5′-tetramethyl benzidine substrate)-ELISA solution (Pierce Biotechnology) until the desired absorbance was reached (5–10 min). The reaction was quenched by the addition of 2 N aqueous H_2_SO_4_ and the absorbance was measured at 450 nm using a BioTek Synergy 2 96-well plate reader. Absorbance was plotted versus concentration (logM) and fit to a non-linear regression analysis using GraphPad Prism 8 software [model: log(inhibitor) vs. response-variable slope (four parameters)]. The calculated IC_50_ values presented in Table 2 are from multiple independent experiments and were normalized and averaged to provide values ± SEM.

## 4. Conclusions

Presented herein are the design of the triazole-containing conformationally constrained peptides **3a**–**3d** and the His mimic-containing peptide **4b** as well as their facile preparation using on-resin azide-alkyne cycloaddition reactions. The resulting peptides were evaluated in FP binding assays using isolated PBDs of Plk1, Plk2 and Plk3 and in ELISA assays against full-length Plk1. Certain of these new ligands retain the high Plk1 PBD-binding affinity of the parent peptide **2a**, while having enhanced selectivity for the PBD of Plk1 relative to the PBDs of Plk2 and Plk3. It is interesting that peptides **4b** and **3d** show significantly greater than anticipated reduced affinities in full-length Plk1 ELISA assays relative to values obtained with the isolated PBD (160-fold for **4b** and 480-fold for **3d**). The larger differences may indicate a reduced ability of these triazole-containing peptides to effectively relieve auto-inhibition arising from interdomain interactions between the KD and PBD or to engage the PBD cryptic pocket in the full-length construct. These observations are noteworthy, in that they potentially indicate structural interactions of the KD and PBD in full-length Plk1 that are not anticipated by the previous co-crystal structure of isolated KD and PBD in the presence of Map205.

## Figures and Tables

**Table 1:**
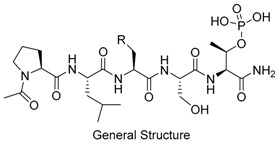
Inhibitory potencies of peptides containing different linkers using a fluorescence polarization (FP) assay. Polo-like kinase (Plk), polo-box domain (PBD).

Peptide	R	IC_50_ (nM)		
		PLK1 PBD	PLK2 PBD	PLK3 PBD
**1** *^i^*	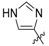	650 ± 39	ND *^iv^*	ND
**17**	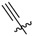	1000 ± 140	ND	ND
**13**	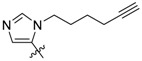	1100 ± 45	ND	ND
**2a** *^ii^*	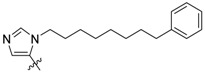	15 ± 0.94	220 ± 15	1100 ± 230
**2b** *^iii^*	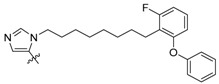	15 ± 0.33	180 ± 14	450 ± 87
**3a**	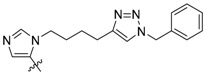	110 ± 6.6	ND	ND
**3b**	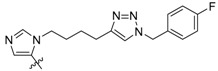	100 ± 19	ND	ND
**3c**	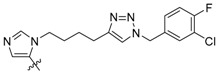	130 ± 9.8	ND	ND
**3d**	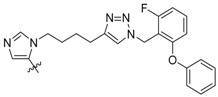	25 ± 1.6	5900 ± 420	9900 ± 2200
**4b**	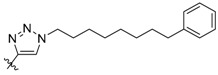	17 ± 0.17	690 ± 20	3400 ± 130

*^i^* See reference [14]; *^ii^* See reference [15]; *^iii^* See references [25,26]; *^iv^* Not determined.

**Table 2:** PBD-binding affinities of peptides against full-length Plk1 using an ELISA assay.

Peptide	IC_50_ (nM)
**2a** *^i^*	400 ± 32 (27×) *^iii^*
**2b** *^ii^*	290 ± 6.7 (19×)
**3d**	12000 ± 2100 (480×)
**4b**	2700 ± 190 (159×)

*^i^* See reference [15]; *^ii^* See references [25,26]; ^*iii*^ Fold-change relative to isolated PBD value.

## Supplementary Materials

Supplementary material associated with this article is available online. Figures S1 and S2 reporting FP binding data against the isolated PBDs of Plk1, Plk2 and Plk3 and ELISA binding assays against full-length Plk1.
