# Supplementary material for: Development of Highly Selective 1,2,3-Triazole-containing Peptidic Polo-like Kinase 1 Polo-box Domain-binding Inhibitors
Source: Molecules. 2019 Apr 16;24(8):1488. doi: 10.3390/molecules24081488 (PMC6515314; doi:10.3390/molecules24081488)
Supplement: Supplementary file 1 [file molecules-24-01488-s001.pdf]

Supplementary Material

Development of Highly Selective 1,2,3-Triazole-containing Peptidic Polo-like Kinase 1 Polo-box Domain-binding Inhibitors

Xue Zhi Zhao*, Kohei Tsuji, David Hymel, Terrence R. Burke, Jr.*

Chemical Biology Laboratory, Center for Cancer Research, National Cancer Institute, National Institutes of Health, Frederick, Maryland 21702, U.S.A.

* Correspondence: xuezhi.zhao@nih.gov (X.Z.Z.) and burkete@nih.gov (T.R.B.Jr.); Tel.: +1-301-846-5907 (X.Z.Z.); +1-301-846-5906 (T.R.B.Jr.)

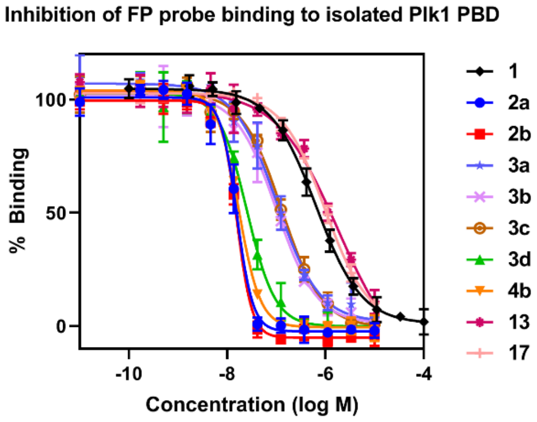

(a)

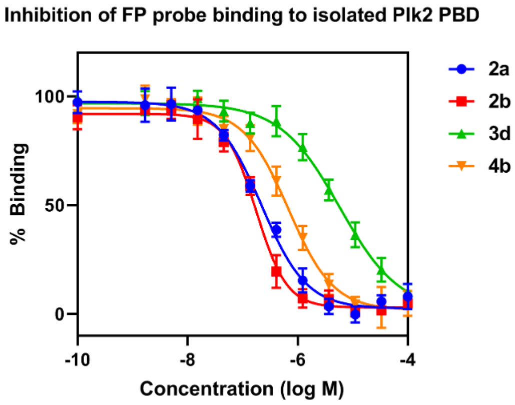

(b)

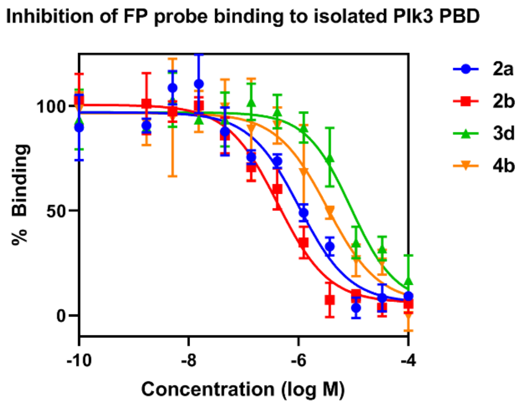

(c)

Figure S1. Binding data obtained from FP assays using the PBDs of Plk1 (a), Plk2 (b) and Plk3 (c).

Experimental procedures are presented in the main text.

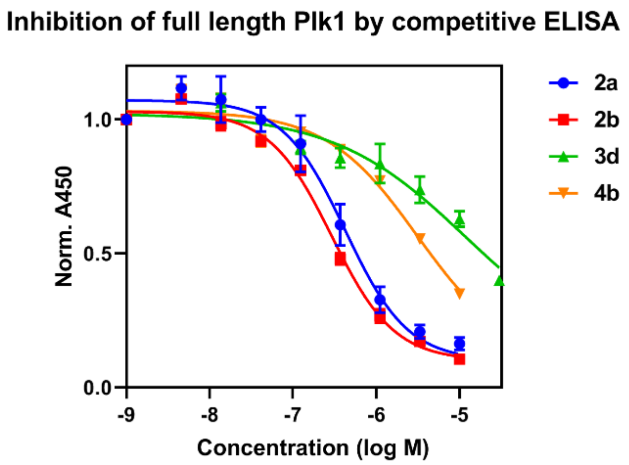

Figure S2. Binding data obtained from ELISA assays using full-length Plk1. Experimental procedures are presented in the main text.
